# Nosocomial Infections in Burned Patients in Motahari Hospital, Tehran, Iran

**DOI:** 10.1155/2011/436952

**Published:** 2011-11-14

**Authors:** Leila Azimi, Abbas Motevallian, Amirmorteza Ebrahimzadeh Namvar, Babak Asghari, Abdolaziz Rastegar Lari

**Affiliations:** ^1^Antimicrobial-Resistance Research Center and Department of Microbiology, Faculty of Medicine, Tehran University of Medical Sciences, P.O. Box 14515-717, Tehran, Iran; ^2^School of Public Health, Tehran University of Medical Sciences, P.O. Box 14515-717, Tehran, Iran

## Abstract

Burn patients are at high risk of developing nosocomial infection because of their destroyed skin barrier and suppressed immune system, compounded by prolonged hospitalization and invasive therapeutic and diagnostic procedures. Studies on nosocomial infection in burn patients are not well described. The objective of the present study was to identify the causative bacterial of nosocomial infection and to determine the incidence of nosocomial infection and their changing during hospitalization in burned patients admitted to in the Motahari Hospital, Tehran, Iran. During the second part of 2010, 164 patients were included in this study. Samples were taken the first 48 hours and the fourth week after admission to Motahari Burn hospital. Isolation and identification of microorganisms was performed using the standard procedure. Of the 164 patients, 717 samples were taken and 812 bacteria were identified, 610 patients were culture positive on day 7 while 24 (17.2%) on 14 days after admission. The bacteria causing infections were 325 *Pseudomonas*, 140 *Acinetobacter*, 132 Staphylococcus aureus, and 215 others. The percentage of mortality was 12%. All of patients had at least 1 positive culture with *Pseudomonas* and/or with *Acinetobacter*. Hospitals suggest continuous observationof burn infections and increase strategies for antimicrobial resistance control and treatment of infectious complications.

## 1. Introduction

Nosocomial infections are one of the most common complications affecting hospitalized patients and contribute to excess morbidity and mortality [1]. Hospitalized patients in burn care wards are at higher risk for hospital-associated infections due to the immunocompromising effects of burn injury [2]. Nosocomial infections are associated with increased length of stay, prolonged therapy, and increased costs [3]. Burn injury is the most important health problem in many countries of the world [4, 5]. Organisms associated with nosocomial infections in burn patients include organisms found in the patient's own endogenous (normal) flora, from exogenous sources in the environment, and from healthcare personnel. The distribution of organisms changes over time in the individual patient and such variation can be improved with suitable management of the burn wound and patient [6].

The dominant flora of burn wounds during hospitalization changes from Gram-positive bacteria such as *Staphylococcus* to Gram-negative bacteria like *Pseudomonas aeruginosa.* The majority of *P. aeruginosa*, an opportunistic human pathogen, isolates from burn patients were multidrug resistant (MDR) [5, 7]. However, different studies have shown that *Staphylococcus aureus* is one of the greatest causes of nosocomial infection in these patients [1, 8]. Previous study in Taleghani Burn Hospital in Khuzestan province, Iran, was carried out to determine nosocomial infections in burned patients [9]. Based on National Nosocomial Infection Surveillance System (NNIS) criteria, all the burned patients are required to follow the distribution of bacterial species among burn isolates [10]. The purpose of this study was to identify the causative bacterial of nosocomial infection and determine frequency of bacterial species and their changing during hospitalization in burned patients admitted to Motahari Burn Center. The purpose of this study was not generalizing the results of this study to the specific population. This study will improve our knowledge about the current epidemiologic situation for a better planning and providing the best possible care to this population of patients. 

## 2. Materials and Methods

In a descriptive study, the incidence of nosocomial infection was calculated on the base of 1000 patient-day. Results were analyzed using SPSS 18, and statistical analysis was performed. The medical records database of the Motahari burn care center was searched to identify 164 patients admitted from second part of 2010. For each admission, the following information was extracted: age, total body surface area burned, injury severity score, length of stay in hospital, length of stay in the ICU, days requiring mechanical ventilation, presence of inhalation injury, and survival to hospital discharge. In addition, the microbiology records were searched to determine which patients had cultures growing microorganisms. Motahari Hospital is the only referral burn center in Tehran. Surveillance of nosocomial infections in burn units should be performed as recommended by the National Nosocomial Infections Surveillance system in Motahari Hospital, Tehran, Iran. In the present study, 164 patients are analyzed, that were 53 females and 111 males. Their age range is between 1and 88 years and all of them hospitalized at least 2 weeks, burns degree at least was II and, in the most of them, TBSA (the total body surface area) was more than 10%. For All of them, topical antiseptic solution and normal saline were used, and the dressings were changed daily. Mupirocin was administered as prophylactic antibiotic. Mupirocin is a topical antimicrobial drug indicated as an adjunct for the prevention and treatment of wound sepsis in patients with second- and third-degree burns. The rationales for the 4-week follow-up duration were found to have active nosocomial infection during this period and effectiveness of antimicrobial therapy. To distinguish the different bacteria from wounds, all samples examined in the same setting and laboratory routine culture media such as Blood Agar, Eosin Methylen Blue, and Nutrient agar were used. In the next step, growth at 37°C in Brain Heart Infusion (BHI), the oxidative and oxidative-fermentation (OF) test for identification of *Pseudomonas* and *Acinetobacter*, and the specific test for detection of Enterobacteriaceae spp. is necessary.

## 3. Results

During the 6-month study period, 164 burn patients were admitted to the hospital. Mean age was 1–100 years. Mean burn level range was (8%–100%). There was no statistically significant correlation between the extent of burn and incidence of infection (*P* ~ .098).

**Figure 1:**
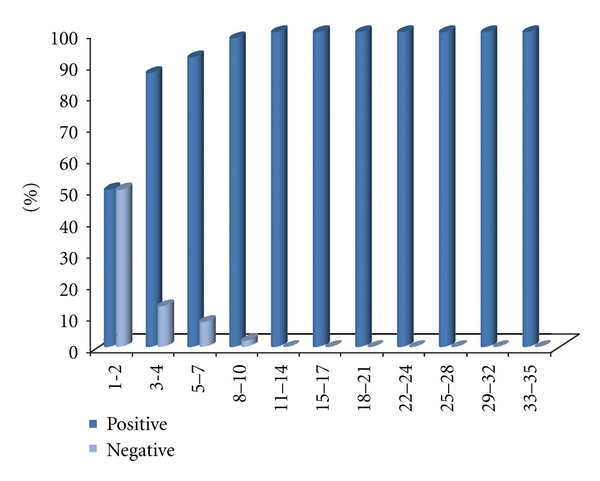
Frequencies of positive and negative culture in different days.

A total of 812 bacterial isolates were obtained. The bacterial isolate was 325 (40%) *Pseudomonas*, 140 (17%) *Acinetobacter*, 132 (16%) *S. aureus*, and 215 (27%) other bacteria. More than one kind of bacteria was identified in 95 samples from 717. 40 percent of cultures were positive without *Pseudomonas* and *Acinetobacter *in first 48 hours after admission. In this study, relationship between positive and negative cultures was statistically significant. Late in the first week 67% of patient had at least one of *Pseudomonas* and/or *Acinetobacter*. This percentage in second, third, and fourth week was 81, 84, and 98%, respectively. 13 samples (29%) of 45 blood cultures were positive (11 with *Pseudomonas* and 2 *Acinetobacter*). Mortality is 12% among patients and all of them had Acinetobacter (3 samples) and *Pseudomonas aeruginosa* and Acinetobacter (7 samples) in their positive culture (Tables 1 and 2, Figure 1).

## 4. Discussion

Nosocomial infections are a significant problem for health services in all countries, with important effects on the survival of high-risk patients, such as burn patients. Infections of burn sites are very dangerous problems that can compromise the patients survival and the outcome of reconstructive treatment [3]. Sufficient research on nosocomial infections in burned patients has not been done. Despite numerous epidemiological studies have been published in burn wound infections in Iran, inadequate data is available on nosocomial infection. The first report of nosocomial infection in a burn hospital in Tehran was achieved in 2000 [7]. According to the CDC protocol [10], *Pseudomonas *and *Acinetobacter* are members of nosocomial microorganism. In some countries such as Iraq, *S. aureus* can be considered as a major cause of nosocomial infection in burn wounds. In this present study, 40% of 164 patients had been positively cultureal without *Pseudomonas* and *Acinetobacter* in the first 48 hours after admission. Replacement of positive cultures and the other hand colonization of negative cultures caused number of *Pseudomonas* and *Acinetobacter* samples reached to 81, 84% in next week and finally 98% in the fourth week. This issue can showed to change different genus and species of bacteria in positive and negative cultures that represent nosocomial infections in burn wound. A nosocomial outbreak of* Acinetobacter* in the burns unit of a university hospital in Toulouse occurred in France in 2004 [11]. In Iraq, *S. aureus* was the most isolated agent [12], but studies from England [13] and Turkey have shown that *Pseudomonas* spp. were more important for isolation [14]. In Sao Paulo *Acinetobacter* was the most isolated from catheter-related infections in burned patients [15]. The mortality rate in these patients was 20 cases (12%) that was 65% of them had third degree burns. All of them at least had one positive culture with *Pseudomonas* and/or *Acinetobacter*. Fortunately, mortality related to burn in burn patients has decreased. According to past studies conducted, this percentage was 19% [7] in Tehran hospital and 34.45% in south west of Iran in 2000 [5, 16].

Hand hygiene and other approaches such as modification of hospital environment may be particularly beneficial strategies to increase control nosocomial infection [17]. Patient characteristics such as age, sex, smoking history, nutritional status, and underlying diseases and conditions of patients such as diabetes, chronic renal, and liver diseases may affect the occurrence of infection in burned patients [18]. In burn patients, the primary means is direct or indirect contact, either via the hands of the staff caring for the patient or from contact with unsuitable decontaminated equipment. Burn patients are unique in their vulnerability to colonization from organisms in the environment as well as in their tendency to disperse organisms into the surrounding environment [6]. In general, the larger burn injury is, the greater the volume of organisms that will be dispersed into the environment from the patient. Appropriate use of diagnostic procedures, invasive devices, and medical therapy, particularly antibiotics, may also decrease the likelihood of nosocomial infections [19]. Prevention of infection in burn patient is an important issue that should be considered in burns unit. Isolation of these patients, health policy such as control of staff and nurses, sterilization of bed sheets, dressing and other equipment related to these patients, and preparation of optimum care conditions of burn patients can be helpful to treat of them. Mupirocin 2% were equally effective in reducing local burn wound bacterial count and preventing systemic infection.

 On the other hand, the antimicrobial pattern of resistance is a very important option for treatment in burn patients. Using new extended-spectrum antibiotic can be useful for treatment. The results of this study increase our epidemiological information about recent situation of burn and prepare the best situation for watchful of these patients population.

## Figures and Tables

**Table 1:** Characterization of 164 patients.

Total number	164	
Male	111	68%
Female	53	32%

Age (yr)		

1–15	25	15%
16–30	59	36%
31–45	40	24%
46–60	23	15%
61–75	13	8%
76–88	4	2%

Range of total body surface area burned		

(Percentage)	64	39%
1–29	51	31%
30–50	16	10%
51–69	14	8%
70–100	19	12%
Electricity		

**Table 2:** Number and kind of bacteria was identified from 164 patients in first hours and other weeks.

	*Pseudomonas*		*Acinetobacter*		*S. aureus*		*Pse + Aci + S*. *aureus*	*Pse + Aci*	*Pse + S*. *aureus*	*Aci + S. aureus*
	Total *Pseudomonas isolated *	Only *Pseudomonas isolated *	Total *Acinetobacter isolated *	Only *Acinetobacter isolated *	Total *S*. *aures isolated *	Only *S*. *aures isolated *				
First 48 hrs	4 (16%)	4 (16%)	1 (4%)	1 (4%)	18 (72%)	18 (72%)	—	—	—	—
Last of first week	49 (55.5%)	33 (34%)	20 (20.6%)	9 (9.2%)	28 (28.8%)	15 (15.4%)	2 (2%)	6 (6.1%)	8 (8.2%)	3 (3.1%)
Second week	101(55.1%)	61 (33.3%)	51 (27.8%)	19 (10.3%)	31 (16.9%)	17 (9.2%)	2 (1.1%)	28 (15.3%)	10 (5.4%)	2 (1.1%)
Third week	59 (64.8%)	37 (40.6%)	17 (18.6%)	2 (2.1%)	15 (16.4%)	5 (5.4%)	1 (1.1%)	13 (14.2%)	8 (8.7%)	1 (1.1%)
Fourth week	49 (58.3%)	26 (30.9%)	23 (27.3%)	6 (7.1%)	12 (14.2%)	1 (1.2%)	2 (2.3%)	15 (17.8%)	9 (10.7%)	—

## References

[1] Emori TG, Gaynes RP (1993). An overview of nosocomial infections, including the role of the microbiology laboratory. *Clinical Microbiology Reviews*.

[2] Mosier MJ, Pham TN (2009). American burn association practice guidelines for prevention, diagnosis, and treatment of ventilator-associated pneumonia (VAP) in burn patients. *Journal of Burn Care and Research*.

[3] Cosgrove SE (2006). The relationship between antimicrobial resistance and patient outcomes: mortality, length of hospital stay, and health care costs. *Clinical Infectious Diseases*.

[4] Lari AR, Alaghehbandan R, Nikui R (2000). Epidemiological study of 3341 burns patients during three years in Tehran, Iran. *Burns*.

[5] Rastegar Lari A, Bahrami Honar H, Alaghehbandan R (1998). Pseudomonas infections in Tohid Burn Center, Iran. *Burns*.

[6] Sharma BR (2007). Infection in patients with severe burns: causes and prevention thereof. *Infectious Disease Clinics of North America*.

[7] Lari AR, Alaghehbandan R (2000). Nosocomial infections in an Iranian burn care center. *Burns*.

[8] Warner P, Neely A, Bailey JK, Yakuboff KP, Kagan RJ (2009). Methicillin-resistant staphylococcus aureus furunculitis in the outpatient burn setting. *Journal of Burn Care and Research*.

[9] Ekrami A, Kalantar E (2007). Bacterial infections in burn patients at a burn hospital in Iran. *Indian Journal of Medical Research*.

[11] Héritier C, Dubouix A, Poirel L, Marty N, Nordmann P (2005). A nosocomial outbreak of Acinetobacter baumannii isolates expressing the carbapenem-hydrolysing oxacillinase OXA-58. *Journal of Antimicrobial Chemotherapy*.

[12] Qader AR, Muhamad JA (2010). Nosocomial infection in sulaimani burn hospital, IRAQ. *Annals of Burns and Fire Disasters*.

[13] Kerr KG, Snelling AM (2009). Pseudomonas aeruginosa: a formidable and ever-present adversary. *Journal of Hospital Infection*.

[14] Oncul O, Ulkur E, Acar A (2009). Prospective analysis of nosocomial infections in a burn care unit, Turkey. *Indian Journal of Medical Research*.

[15] Campos Jú CP, Sanches P, Tedokon EA, Souza ACR, Machado RLD, Rossit ARB (2010). Catheter-related infections in a northwestern São Paulo reference unit for burned patients care. *Brazilian Journal of Infectious Diseases*.

[16] Panjeshahin MR, Lari AR, Talei AR, Shamsnia J, Alaghehbandan R (2001). Epidemiology and mortality of burns in the South West of Iran. *Burns*.

[17] Pittet D, Hugonnet S, Harbarth S (2000). Effectiveness of a hospital-wide programme to improve compliance with hand hygiene. *The Lancet*.

[18] Graninger W, Ragette R (1992). Nosocomial bacteremia due to Enterococcus faecalis without endocarditis. *Clinical Infectious Diseases*.

[19] Craven DE, Steger KA, Barber TW (1991). Preventing nosocomial pneumonia: state of the art and perspectives for the 1990s. *American Journal of Medicine*.

